# Flipping the tanycyte switch: how circulating signals gain direct access to the metabolic brain

**DOI:** 10.18632/aging.100557

**Published:** 2013-05-11

**Authors:** Vincent Prevot, Fanny Langlet, Benedicte Dehouck

**Affiliations:** Laboratory of Development and Plasticity of the Postnatal Brain, Jean-Pierre Aubert Research Centre (JPARC), Inserm U837, University of Lille 2, Lille, 59000, France

The survival of an organism relies on its ability to promptly, effectively and reproducibly communicate with brain networks that control food intake and energy homeostasis [1, 2]. Although the access of most circulating factors to the brain requires transcellular transport across the blood-brain barrier, certain hypothalamic areas that play a critical role in the control of appetite and body weight, such as the arcuate nucleus of the hypothalamus (ARH), might require direct access to peripheral homeostatic signals by a privileged route that bypasses brain barriers. The question as to whether such a route exists and can be modulated to meet physiological demands in response to changes in feeding status is largely unexplored, and represents an issue central to our understanding of the mechanisms controlling energy balance and metabolism [3]. In a recent study, we have established a new physiological concept in the regulation of energy homeostasis by showing that the nutritional status of an individual modulates the permeability of discrete blood-hypothalamus barriers to circulating metabolic signals, permitting them to directly access a subset of ARH neurons [4].

**Figure 1:**
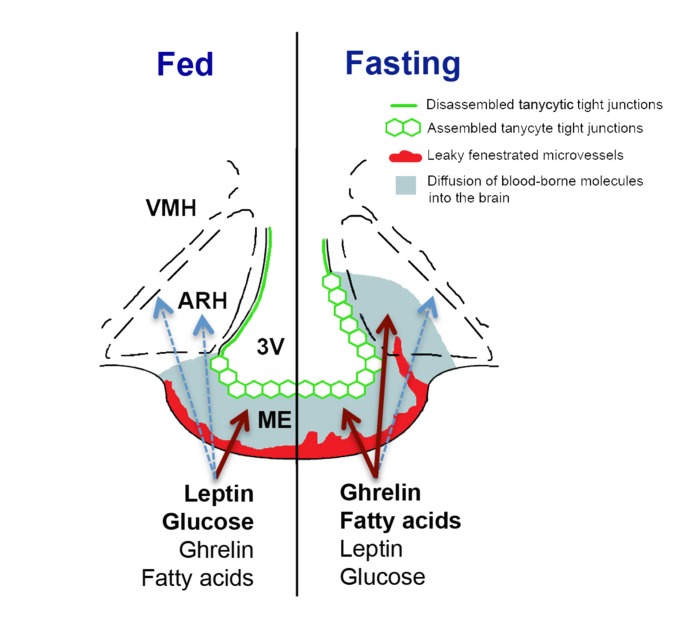
Schematic diagram illustrating structural differences between the median eminence and ARH of mice fed *ad libitum* and fasting mice, and their effects on the diffusion of blood-borne signals into the brain The arcuate nucleus of the hypothalamus (ARH) lies lateral to the third ventricle (3V) and immediately dorsolateral to the median eminence (ME). In mice fed normally (left half of the figure), the fenestrated blood vessels of the ME permit the local diffusion of macromolecules from the circulation, while vessels in the ARH proper exhibit blood-brain barrier properties that block this diffusion (not shown). Hence, circulating metabolic signals whose levels are high in the fed state (e.g., leptin and glucose) require BBB transport to access ARH neurons. Under these conditions, tight junctions (green) between tanycytes line the ventricular wall of the ME, preventing the diffusion of circulating factors into the 3V and CSF. During fasting or energy restriction (right half of the figure), the levels of hormones such as ghrelin rise, along with products of lipolysis (e.g., fatty acids), while leptin and glucose levels fall. Concomitantly, some ME vessels extending into the ARH become fenestrated, while the tight junction barrier along the 3V extends dorsally. These changes allow the freer diffusion of circulating signals that indicate energy restriction to ARH cells, including AgRP/NPY neurons that lie in the ventromedial ARH, while preventing the access of these substances to the rest of the brain through the CSF. The focal plasticity of this dual-faceted blood-hypothalamus barrier thus enhances the orexigenic/anabolic response to energy deficits.

Many metabolic activities are coordinated by neurons of the ARH [1, 2]. This regulation is primarily mediated by the balance between anorexigenic neurons expressing proopiomelanocortin (POMC) and orexigenic neurons expressing neuropeptide Y (NPY) and Agouti-related protein (AgRP). For example, under fasting conditions, decreased systemic levels of nutrients (e.g. glucose) and changes in related hormone levels (e.g. decreased leptin and increased ghrelin) act to activate NPY/AgRP neurons and inhibit POMC neurons in the hypothalamus. This leads to a marked anabolic state within the hypothalamus that translates into a potent stimulus for the animal to seek and ingest food. The ARH lies adjacent to the median eminence, which contains a blood-cerebrospinal fluid (CSF) barrier (see Figure 1) composed of tanycytes, specialized hypothalamic glia that line the floor of the third ventricle and extend processes to contact a specialized capillary plexus [4]. The endothelial cells of this median eminence capillary plexus are unique in being fenestrated and their permeability allows for the passive and rapid extravasation of the majority of nutrients and metabolic hormones circulating in the pituitary portal blood (i.e., with molecular sizes bellow 20 kDa) [5]. However, the restriction of this capillary fenestration to the median eminence [4] together with the occurrence of tight junction complexes between adjacent tanycytes that act as a physical barrier [4], sequesters these molecules within the median eminence and prevents their diffusion to the rest of the brain via the CSF in animals fed ad libitum [4] (Figure 1). In contrast, in fasting mice, dips in blood glucose levels, likely perceived by tanycytes themselves thanks to their glucosensing properties [6], trigger VEGFA expression in these cells, and VEGF accumulation in the median eminence acts on endothelial VEGFR2 to promote the fenestration of capillary loops that reach the ARH [4]. In consequence, some target neurons in the ventromedial ARH are no longer insulated by the blood-brain and blood-CSF barriers but become directly exposed to peripheral metabolic signals, a process that is aided by the sealing of the paracellular space between the parenchyma and the CSF by the reorganization of the tight junction complexes of tanycytes that contact these newly permeable microvessel loops (Figure 1) [4]. This situation is reversed upon refeeding. The increased accessibility of the ARH during fasting is confirmed both by the leakage of intravenously injected dye into the ventromedial ARH [4] and by the increased binding of blood-borne fluorescently-labeled ghrelin, an orexigenic hormone, to NPY/AgRP neurons [5], a phenomenon associated with a hyperphagic response upon refeeding [4]. Together, these findings suggest that VEGF-mediated structural changes at the brain-hypothalamic barrier, by modulating the access of blood-borne metabolic substrates to the ARH, play an important role in the adaptive response to fasting.

The median eminence is not the only brain region to exhibit this kind of gliovascular organization [7]. For example, both fenestrated capillaries and neurons capable of sensing metabolic signals are found in the brainstem region containing the area postrema. Whether nutritional status modulates the plasticity of blood-CSF barriers in this or other regions needs to be determined in future studies. Similarly, the pathophysiological implications of this reorganization of endothelial fenestration/tight junction complexes also remain to be explored. For instance, are these fasting-induced blood-hypothalamic barrier changes altered in animal models of diet-induced hormone resistance? Is VEGF-A signaling involved in the control of physiological and/or high-fat-diet-induced neurogenic activity in hypothalamic tanycytes [6]? Is the attenuated response to fasting in aged animals [8] associated with altered vascular/glial reorganization in the median eminence? And finally, could this VEGFdependent brain-hypothalamus barrier plasticity hold therapeutic potential for treating dysfunctions of the neuroendocrine control of energy homeostasis in hormone-resistant individuals [8]? The piecing together of these and other parts of the metabolic puzzle promises to be quite exciting!
